# 10 Principles of Geriatric Psychiatry

**DOI:** 10.1007/s40596-025-02126-6

**Published:** 2025-03-03

**Authors:** Leah G. Richler, Mariko Shimizu, Rachel Kester

**Affiliations:** 1 UMASS Chan Medical School/UMass Memorial Healthcare, Worcester, MA USA; 2 Cambridge Health Alliance, Cambridge, MA USA

Geriatric psychiatry is one of the youngest medical specialties in the USA. The American Association of Geriatric Psychiatry was founded in 1978, and the American Board of Psychiatry and Neurology offered the first certification examination in 1991 [1]. Since that time 3754 geriatric psychiatry certificates have been awarded. As of 2023, there were only 1429 board-certified geriatric psychiatrists [2].

Clearly, non-geriatric psychiatrists such as primary care providers and general adult psychiatrists will provide most of the mental health care for this population. Knowing this, training general psychiatry residents in the principles of geriatric psychiatry is critical to allow them to care for this population and educate their colleagues.

According to the Accreditation Council of Graduate Medical Education Program Requirements for Graduate Medical Education in Psychiatry [3], effective July 1, 2022, “Resident experience in geriatric psychiatry must include one-month full time equivalent of organized experience focused on areas unique to the care of the elderly.” Although this language allows for flexibility for training opportunities depending on availability of resources at each institution, a systematized approach to teaching principles of care can be helpful. Certain overriding principles and skills apply across all clinical care settings and can help guide educators as they train residents. While educating residents and colleagues, offering explicit and focused principles as a framework for providing care can be grounding during the training years (Table 1).

**Table 1:** Framework for providing care in geriatric psychiatry

Principle	Key points
1. Honor the uniqueness of every older adult	Geriatric population is a highly heterogeneous group Divergence between chronological and biological age becomes more pronounced with aging Recognition and evaluation of frailty is crucial
2. Do not change more than one thing at a time	It is sound scientific practice to change one variable at a time Psychoeducation on realistic expectations of intervention is helpful for older adults and their caregivers Close observation and watchful waiting are a legitimate and thoughtful clinical decision
3. Start low, go slow, but do not stop	Changes in pharmacodynamics, pharmacokinetics, absorption, metabolism, and excretion of medications must be considered Efficacy of medications can be limited in older adults due to metabolic abnormalities and structural brain changes There is a tendency to maintain low and ineffective doses of medications, resulting in undertreatment and polypharmacy
4. Polypharmacy is always on the differential diagnosis	Older adults are at risk of polypharmacy Careful medication review and consolidation can effectively avoid overprescribing Deprescribing can reduce symptoms and improve function
5. Prioritize safety	Safety is the highest priority for older adults and those around them Assessment of activities of daily living and of instrumental activities of daily living is an essential part of a geriatric safety evaluation Older adults and caregivers may benefit from referrals to appropriate community services to help them remain in the community as long as possible Caregivers may need a reminder to contact emergency services in case of emergency
6. Assess functioning	Non-psychiatric causes of functional impairment, including sensory, cognitive, and motor difficulties, must be carefully examined Non-psychiatric conditions can greatly impact severity and response to psychiatric treatment
7. Assume capacity until proven otherwise	Having a diagnosis of dementia does not automatically translate into lack of medical decisional capacity Capacity evaluation is affected by the complexity, risks, and benefits of each medical decision to be made
8. Plan for the future now	Advanced care planning clarifies patients’ value, wishes, and preferences in their medical care and is associated with improved outcomes, including satisfaction with care and fewer hospitalizations Planning should take place as early as possible
9. Engage colleagues	The complexity of needs of older adults necessitates multidisciplinary care within and outside of health care systems Collateral information from multiple sources allows clinicians to gain more comprehensive and realistic understanding of older adults
10. Change is possible	While certain aspects of cognition may decline with age, older adults continue to develop strengths and are able to adopt changes Society can reflect on and change its biases and beliefs on aging

## Principle 1: Honor the Uniqueness of Every Older Adult

Although the geriatric population is often seen as a homogenous group, variability in this demographic is vast. In practice, people in their 60 s may present with significant physical and cognitive impairment requiring maximal support, and people in their 90 s can remain physically and cognitively healthy and functionally independent. The divergence between chronological and biological age becomes more apparent with age. In part, this is due to the complex interplay of biological, psychological, and social factors which influences physiologic reserve.

Like those who practice geriatric medicine, geriatric psychiatry educators must teach learners to evaluate frailty, defined as a state of increased vulnerability across multiple health domains that leads to adverse health outcomes in order to manage symptoms and optimize functioning [4]. Therefore, aging can be seen through the lens of reserve loss. In geriatric psychiatry there is a focus on physical, cognitive, and functional status, all of which can vary independently and in relation to one another. There is value in screening for and calculating a frailty index to gauge the overall risks of the patient with respect to loss of independence and disability. There are several indices and calculators used, but it is preferable to teach learners to utilize a simple screening tool and then a more comprehensive calculator if appropriate [5].

## Principle 2: Do Not Change More Than One Variable at a Time

Although changing one variable at a time is generally good clinical practice, it can be difficult to achieve in reality. Helping learners to provide patients and their families with realistic expectations of interventions is as important as the results of the interventions themselves.

Medicine in general, and psychiatry in particular, takes into account the larger biopsychosocial context in which people live, understanding that there are many complicated variables at play. In this population, not only are providers making changes but also patients themselves are moving targets. When prescribing psychiatric medications in this population, going slowly, and adjusting no more than one variable at a time, is key to understanding cause and effect. Though there is a tendency to focus on medications as a major variable, it is important to remember that factors other than medications can result in change. Without provider interventions, changes can occur organically. Medical and neurologic comorbidity and lifestyle modifications all can independently result in physiologic changes and affect the larger presentation of the individual. Therefore, sometimes, the best approach is close observation and watchful waiting. Providing reassurance of this fact to learners can be incredibly valuable.

## Principle 3: Start Low, Go Slow, But Do Not Stop

Physiologic changes that occur with aging affect both pharmacodynamics (i.e., what the medication does to the body) and pharmacokinetics (i.e., what the body does to the medication). Absorption, metabolism, and excretion of medications differ in older adults. Also, the likelihood of polypharmacy and medication-to-medication interactions is higher in the elderly. It is critical to remind learners to consider these factors when prescribing medications in this population to avoid adverse effects and morbidity [6].

Older adults are also more likely to develop an intolerable side effect, and often medication side effects mimic underlying symptomatology. Though medications can be helpful and even lifesaving in some situations, in those adults with metabolic abnormalities and structural brain differences, their efficacy is limited [7]. Also, given the increased sensitivity of those with limited physiologic reserve, the risk of side effects often outweighs the potential benefit. Because of possible adverse side effects, there is a tendency to maintain low and ineffective doses resulting in undertreatment and polypharmacy.

## Principle 4: Polypharmacy Is Always on the Differential Diagnosis

The likelihood of symptoms being related to medication side effects is high. Polypharmacy is typically defined as taking five or more medications on a regular basis. Aging adults are at risk for polypharmacy, given their medical complexity. In a 2019 report [8], the percentage of US adults aged 40–79 who used more than five prescription medications within the last 30 days was more than 22.4%. The more complex the medication regimen becomes, the greater the risk of error in administration leading to adverse events and reactions. Sixty-nine percent of medications have between 10 and 100 different side effects, based on marketing material [9].

Geriatric psychiatry educators must empower learners to engage in deprescribing with the goal of reducing symptoms and improving function. Educators should help trainees to avoid overprescribing medications regularly and each time a new medication is added. Before prescribing or renewing a medication, consider life expectancy, time to achieve therapeutic benefit, and goals of treatment.

## Principle 5: Prioritize Safety

There is a tendency for physicians to focus on cognitive and non-distressing psychiatric symptoms. For patients and families, functional capacity and safety are often much more relevant considerations. Given the psychiatric and medical complexity of this population, it can become difficult to assess the situation as a whole, as opposed to focusing on individual and treatable symptoms.

Teaching trainees to assess an individual’s ability to maintain activities of daily living and instrumental activities of daily living as part of their safety evaluation is important, paying particular attention to cooking, driving, medication management, and home environment [10]. The Katz Index [11] and Lawton Instrumental Activities of Daily Living Scale [12] provide simple tools to evaluate theses.

Multiple services are available to help patients and their families maintain their autonomy as much as possible in the community. When, even with those services, patients are no longer able to remain safe, health care professionals must help determine an appropriate level of care or support. No medication treats “wandering” or, more generally, the inability to remain safe in the community. Reminding learners that all too often interactions with older people are colored by biases related to aging can help ensure that health care professionals and caretakers maintain considerations of their vulnerability and safety.

## Principle 6: Assess Functioning

The majority of diagnostic criteria in the *Diagnostic and Statistical Manual of Mental Disorders* [13] include an assessment of the ability to function. In order to diagnose someone with a psychiatric disorder, there must be a clinically significant degree of functional impairment. The other requirement is that the symptoms are not better explained by non-psychiatric causes [13]. These non-psychiatric causes can include sensory, cognitive, and motor difficulties. Specific examples include musical ear syndrome, Charles-Bonnet syndrome, subacute delirium, aphasias, bradykinesia, bradyphrenia, and pain. Though such difficulties do not preclude psychiatric illness, they greatly impact the severity and response to psychiatric treatment. Older people are more likely to have these difficulties, which can further exacerbate their comorbid psychiatric illnesses [14]. Helping learners maintain a broad differential and evaluate functional impairment allows for more comprehensive care.

## Principle 7: Assume Capacity Until Proven Otherwise

Psychiatrists are often consulted when a patient’s decisional capacity is in question. Geriatric psychiatry educators in particular must teach learners to understand the ways in which cognition relates to but does not define decisional capacity.

The capacity to make one’s own decisions is essential and integral to autonomy. The ability to make and express a decision is related to, but not explicitly determined by, cognition. Cognition is a broad concept, encompassing knowledge acquisition, complex attention, executive attention, learning and memory, language, perceptual motor function, and social cognition [13]. Dementia, by definition, affects cognition, but the specific areas and degrees of impairment do not necessarily lead to lack of decisional capacity. Further determination of capacity will be impacted by the complexity of the decision, as well as the risks and benefits associated with the decision. Studies have indicated the preserved decisional capacity of older adults with cognitive impairment [15].

## Principle 8: Plan for the Future Now

Advanced care planning allows discussions around patients’ health care management needs to ensure their values, wishes, and preferences are respected and aligned with the medical decisions. It creates a shared understanding among the patient, their caregivers, and their treatment team to facilitate the decision-making process in the complex medical system. Advanced care planning is known to increase patients’ and caregivers’ satisfaction with care and produce optimal health care outcomes including fewer hospitalizations [16].

Given the complexity and potential frailty of this patient population, the likelihood of changes in circumstances and function is high. It is important to begin these conversations by discussing goals of care and planning as early as possible (and adapting over time as necessary). Trainees should be exposed to advanced care planning and should work to become comfortable discussing these issues. Educators must encourage trainees to discuss with patients their preferences and concerns as early as possible and to include in this discussion review of likely challenges and potential outcomes.

## Principle 9: Engage Colleagues

The breadth and complexity of the biopsychosocial needs of older adults means that no single discipline or individual can adequately provide care. In balancing what is desired or ideal with what is possible, given available tools, there is a necessity to involve a multidisciplinary team. Such a team might include, but is not limited to, physicians of varying specialties (e.g., psychiatry, geriatrics, primary care, neurology, palliative care), physician assistants, nurse practitioners, nurses, social workers, pharmacists, personal caregivers, nutritionists, occupational therapists, physical therapists, speech specialists, psychologists, and, most importantly, patients and their loved ones [17]. Other relevant community access points, such as elder services, elder protective services, councils on aging, senior centers, and day programs, play a key role as well.

Obtaining collateral from these resources is important for the clinicians in providing a comprehensive and realistic understanding of an older adult. Multidisciplinary team members should lean on one another’s expertise to promote the overall health of an older adult. Including trainees in these teams and allowing them to learn from all disciplines is integral to their education.

## Principle 10: Change Is Possible

Showing and teaching learners that older adults remain capable of change can alter perspective and management. It is a common misconception that older adults cannot learn new things. In reality, though aging may lead to changes in cognition, including slowing of processing speed and difficulty with multitasking, it does not preclude learning new tasks or improving performance in particular areas [18]. In fact, people who have lived longer may have more experiences to draw on for strength. Various studies have shown that older adults can adopt healthier lifestyles with guidance and support, leading to better functioning [19]. Effort must be made at an individual, community, and societal level to reflect on biases and enhance understanding of aging, with an eventual goal of reframing beliefs on age and aging.

## Conclusion

Regardless of future career path or training, every psychiatrist will encounter older adults as patients, the families of older adult patients, their own aging family members, and, if fortunate enough, their own aging selves. It is one of the missions of geriatric psychiatry as a field to empower its current and future colleagues to care for this unique and important population with skill and respect.

## References

[1] Grossberg GT. Geriatric psychiatry–an emerging specialty. Mo Med. 2010;107(6):401–5. 21319689 PMC6188242

[2] Board of Psychiatry and Neurology. 2023 Annual Report. https://abpn.org/wp-content/uploads/2024/04/ABPN_2023_Annual_Report_WEB.pdf. Accessed 19 May 2024.

[3] Accreditation Council for Graduate Medical Education. Program requirements for graduate medical education in geriatric psychiatry. https://www.acgme.org/globalassets/pfassets/programrequirements/407_geriatricpsychiatry_2022v2.pdf. Accessed 19 May 2024. 10.1055/s-0044-1791821 PMC11669440 39721577

[4] Allison R, Assadzandi S, Adelman M. Frailty: evaluation and management. Am Fam Physician. 2021;103(4):219–26. 33587574

[5] Walston J, Buta B, Xue QL. Frailty screening and interventions. Clin Geriatr Med. 2018;34(1):25–38. 29129215 10.1016/j.cger.2017.09.004 PMC5726589

[6] ElDesoky ES. Pharmacokinetic-pharmacodynamic crisis in the elderly. Am J Ther. 2007;14(5):488–98. 17890940 10.1097/01.mjt.0000183719.84390.4d

[7] Schulz P. Opportunities and challenges in psychopharmacology. Dialogues Clin Neurosci. 2019;21(2):119–30. 31636486 10.31887/DCNS.2019.21.2/pschulz PMC6787536

[8] Hales CM, Servais J, Martin CB, Kohen D. Prescription drug use among adults aged 40–79 in the United States and Canada. August 2019. NCHS Data Brief No. 347. https://www.cdc.gov/nchs/data/databriefs/db347-h.pdf. Accessed 13 Feb 2025. 31442200

[9] Zhang P, Wang F, Hu J, Sorrentino R. Exploring the relationship between drug side-effects and therapeutic indications. AMIA Annu Symp Proc AMIA Symp. 2013;2013:1568–77. 24551427 PMC3900166

[10] Alzheimer’s Association. Safety. https://www.alz.org/help-support/caregiving/safety. Accessed 13 Feb 2025.

[11] Wallace M, Shelkey M, Hartford Institute for Geriatric Nursing. Katz index of independence in activities of daily living (ADL). Urol Nurs. 2007;27(1):93–4. 17390935

[12] Graf C. The Lawton instrumental activities of daily living scale. Am J Nurs. 2008;108(4):52–62; quiz 62–3. 10.1097/01.NAJ.0000314810.46029.74 18367931

[13] American Psychiatric Association. Diagnostic and statistical manual of mental disorders: DSM-5. 5th ed. Washington: American Psychiatric Association; 2013.

[14] Zhang S, Wang Q, Wang X, Qi K, Zhou Y, Zhou C. Longitudinal relationship between sensory impairments and depressive symptoms in older adults: the mediating role of functional limitation. Depress Anxiety. 2022;39(8–9):624–32. 35543591 10.1002/da.23266

[15] Kim SYH, Karlawish JH, Kim HM, Wall IF, Bozoki AC, Appelbaum PS. Preservation of the capacity to appoint a proxy decision maker: implications for dementia research. Arch Gen Psychiatry. 2011;68(2):214. 21300949 10.1001/archgenpsychiatry.2010.191 PMC3349937

[16] Richler LG, Shimizu M, Huang H, Kester R. Challenges of agitation in dementia: a plea for early discussion. Harv Rev Psychiatry. 2023;31(1):22–7. 36608080 10.1097/HRP.0000000000000352 PMC9855747

[17] Tinetti M, Huang A, Molnar F. The Geriatrics 5M’s: a new way of communicating what we do. J Am Geriatr Soc. 2017;65(9):2115. 28586122 10.1111/jgs.14979

[18] National Institute on Aging. 2023. How the aging brain affects thinking. https://www.nia.nih.gov/health/brain-health/how-aging-brain-affects-thinking. Accessed 19 May 2024.

[19] National Institute on Aging. 2020. Cognitive health and older adults. https://www.nia.nih.gov/health/brain-health/cognitive-health-and-older-adults. Accessed 19 May 2024.

